# Mechanisms and immune crosstalk of neutrophil extracellular traps in response to infection

**DOI:** 10.1128/mbio.00189-25

**Published:** 2025-04-16

**Authors:** Qi Liu, Ruke Chen, Ziyan Zhang, Zhou Sha, Haibo Wu

**Affiliations:** 1 School of Life Sciences, Chongqing University 540177, Chongqing, China; The Ohio State University, Columbus, Ohio, USA

**Keywords:** neutrophil extracellular traps (NETs), bacterial infection, evasion, neutrophil, innate immunity, adaptive immunity

## Abstract

Neutrophil extrusion of neutrophil extracellular traps (NETs) in a process called NETosis provides immune defense against extracellular bacteria. It has been observed that bacteria are capable of activating neutrophils to release NETs that subsequently kill them or at least prevent their local spread within host tissue. However, existing studies have mainly focused on the isolated function of NETs, with less attention given to their anti-bacterial mechanisms through interactions with other immune cell populations. The net effect of these complex intercellular interactions, which may act additively, synergistically, or antagonistically, is a critical determinant in the outcomes of host–pathogen interactions. This review summarizes the mechanisms underlying classic NET formation and their crosstalk with the immune system, offering novel insights aimed at balancing the anti-microbial function with their potential inflammatory risks.

## INTRODUCTION

Neutrophils, the dominant immune cells in human blood, form the first line of cell-mediated immunity against various microorganisms. Under homeostatic conditions, neutrophils circulate through the bloodstream and surveil microbial invasion in the body while simultaneously playing integral roles in diverse physiological processes, such as immune cell recruitment, angiogenesis, coagulation, as well as tissue repair (1). Their precursor cells gradually differentiate into promyelocytes in the bone marrow before maturing into functional neutrophils (2). Neutrophils normally circulate in the peripheral blood in a quiescent state (3). Upon infection, endothelial cells capture the circulating neutrophils, guiding their transendothelial migration and chemotaxis to the infection site, where they release inflammatory mediators to initiate immune responses (2). Neutrophils are equipped with a multifaceted arsenal to combat invading pathogens, such as phagocytosis, degranulation, secretion of extracellular vesicles, and formation of neutrophil extracellular traps (NETs) (4, 5). NETs are web-like fibers composed of decondensed chromatin mixed with neutrophil granule content that exhibit potent anti-microbial activity. Defective NET function may facilitate pathogens’ ability to evade the immune system and establish a persistent niche (6).

Emerging evidence highlights the substantial immunoregulatory functions of NETs and their derived proteins. These structures not only play a pivotal role in modulating the inflammatory response but also significantly influence the bactericidal activities of diverse immune cell populations. During pathogen invasion, NETs exert both synergistic and antagonistic effects on immune responses, thereby contributing to the dynamic regulation of host defense mechanisms. Most studies on host–pathogen interactions typically rely on isolated immune cell models for evaluation (7). However, the role of NETs in the immune system is paradoxical. If it is dysregulated, the components of NETs may transform into autoantigens, potentially driving the onset of chronic inflammatory and autoimmune disorders (8). In this review, we first examine the process and functional consequences of NET formation, elucidate the intricate interactions between NETs and other immune cells during bacterial infections, and explore the deleterious effects of NETs that, despite being a critical component of the innate immune response, contribute to pathological outcomes.

## NEUTROPHIL EXTRACELLULAR TRAPS

### Definition, composition, and characteristics of NETs

NETs were first characterized in 2004 as a unique anti-microbial defense mechanism wherein neutrophils expel DNA structures to entrap and partially neutralize pathogens (9). They represent a novel form of programmed cell death, markedly distinct from apoptosis, necroptosis, and pyroptosis, known as NETosis. NETs are web-like DNA structures decorated with histones (H1, H2A, H2B, H3, and H4), along with granule-derived proteins such as neutrophil elastase (NE), myeloperoxidase (MPO), cathepsin G, lactoferrin, and bactericidal permeability-increasing protein, among others (5, 10). While the majority of the DNA within NETs is derived from the nucleus, these extracellular structures also encompass mitochondrial DNA (8). This distinctive characteristic allows NETs to immobilize pathogens within a matrix enriched with anti-microbial proteins. Intriguingly, neutrophils seem particularly adept at sensing microbial size, with NETs proving especially effective against large pathogens that cannot be cleared through phagocytosis, such as *Candida albicans* hyphae and *Mycobacterium bovis* aggregates (11), which proves that NETs are fundamental in combating large microbes *in vivo*. Not all neutrophils within a population are equally susceptible to NET formation. Only activated neutrophils produce NETs, while naïve neutrophils do not. Studies have reported that only a proportion of cells may generate NETs at any given time point (5, 12). Potentially, it could be a safety mechanism to avoid an excessive inflammatory response.

### Induction of NET formation

Neutrophils undergo NETosis in response to various stimuli, including phorbol 12-myristate 13-acetate (PMA), cytokines, autoantibodies, cholesterol crystals, platelets, and various microbial agents (e.g., bacteria, fungi, parasites, and viruses), along with their virulence factors (8). However, the nature of the stimulus detected by neutrophils dictates the specific mode of NET release (13). The β-glucan on the *C. albicans* cell wall is recognized by the dectin-1 receptor on neutrophils, which triggers NETosis (14). The molecular composition of NETs also exhibits stimulus-specific signatures, with distinct proteomic profiles emerging in response to discrete eliciting stimuli. For instance, NETs induced by non-mucoid and mucoid *Pseudomonas aeruginosa* contain 33 common proteins, along with up to 50 variable proteins (15). This complicates direct comparisons across different studies. In addition, NET formation exhibits dose-dependent dynamics, a phenomenon corroborated by recent studies investigating NETs in response to various morphotypes of *Trichophyton rubrum* (16). Furthermore, when multiple stimuli act concurrently on neutrophils, they do not exhibit a synergistic effect; rather, such combined stimuli may actually suppress NET formation (17). Therefore, the induction of NETs demonstrates a pronounced stimulus-dependent multifaceted effect, with their formation being intricately regulated by multiple regulatory mechanisms.

### Mechanisms of NET formation

**Fig 1:**
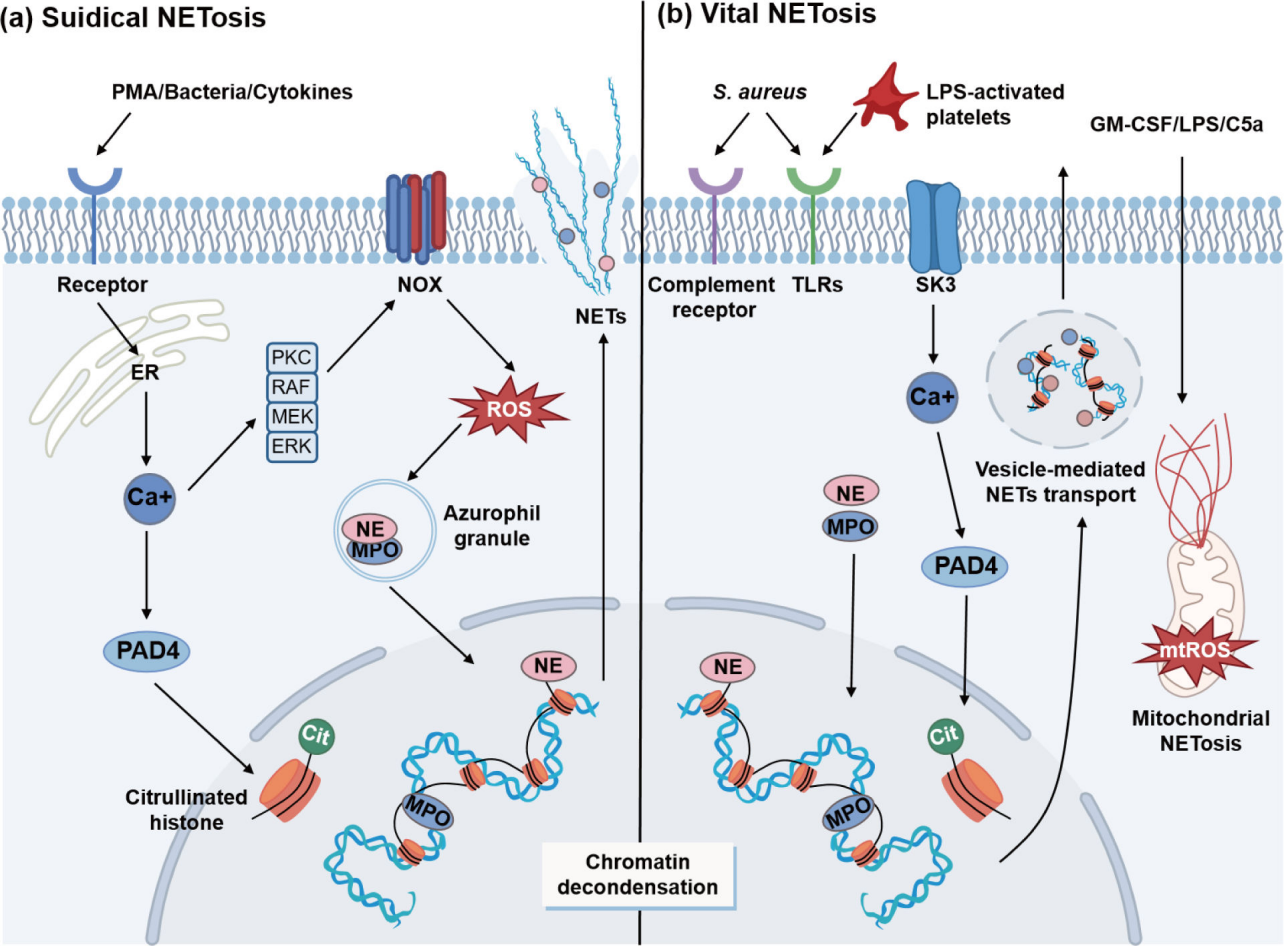
NET formation mechanisms. NET formation can be primarily categorized into two types. The first type is (a) suicidal NETosis, which is initiated by PMA, bacteria, and various cytokines. These extracellular signaling events activate the NADPH oxidase complex (NOX) and subsequently lead to reactive oxygen species (ROS) burst, promoting granule cleavage. The activation of PAD4 and the translocation of MPO and NE into the nucleus contribute to the citrullination of histones and chromatin decondensation. Ultimately, NETs are released and cause neutrophil death. An alternative mechanism, termed (b) vital NETosis, occurs in a NOX-independent manner. Upon interaction with *Staphylococcus aureus* or lipopolysaccharide (LPS)-activated platelets, receptors trigger the influx of calcium ions through the small conductance potassium channel member three (SK3) channel, which activates PAD4. Afterward, the decondensed chromatin coated with anti-microbial proteins is expelled via vesicles without plasma membrane disruption. Additionally, following pretreatment with granulocyte-macrophage colony-stimulating factor (GM-CSF) and subsequent stimulation with LPS or C5a, the mitochondria of neutrophils also exhibit NET formation while retaining neutrophils’ normal physiological functions. Abbreviations: ER, endoplasmic reticulum; MPO, myeloperoxidase; NE, neutrophil elastase; PMA, phorbol 12-myristate 13-acetate; PAD4, peptidylarginine deiminase 4.

Currently, the principal mechanisms underlying NETosis have been classified into two distinct types: suicidal and vital mechanisms (Fig. 1).

#### Suicidal NETosis

Suicidal NETosis was initially identified in neutrophils activated by PMA. Upon activation of cell surface receptors by external stimuli, calcium ions released from the endoplasmic reticulum bind to and activate protein kinase C (PKC). The PKC and RAF-MEK-ERK signaling cascade contribute to the assembly and activation of the NADPH oxidase complex (NOX) (18), leading to a burst of reactive oxygen species (ROS). Elevated ROS levels trigger the activation and translocation of MPO and NE from azurophilic granules to the nucleus, where they facilitate chromatin decondensation and histone cleavage (8). Meanwhile, peptidylarginine deiminase 4 (PAD4), a calcium-dependent enzyme that converts arginine into citrulline, is activated by calcium ions in the cytoplasm (1). The citrullination of histones by PAD4 can potentially modify their protein structure by disrupting the positive charge interactions between histones and DNA, thus promoting chromatin decondensation. Additionally, NE cleaves gasdermin D (GSDMD) in the cytoplasm to generate the 30 kDa NH2-terminal pore-forming fragment, which creates pores in the plasma membrane (19). This ultimately results in cell membrane rupture and death, typically occurring between 2 and 5 hours after the initial stimulus.

Suicidal NETosis does not always rely on NADPH-mediated oxidative bursts. Subsequent studies have shown that calcium ionophores (A23187) and ionomycin can induce this process through a mechanism independent of both NADPH and nitric oxide (NO). Notably, it does not involve the typical NE and MPO but is instead primarily driven by PAD4 (20). Concurrently, calcium modulates mitochondrial ROS (mtROS) production by activating the small conductance potassium channel member three (SK3) (21). mtROS generation is critical for non-NO-dependent NETosis, especially in the absence of functional NADPH oxidase, where they alone are sufficient to drive NET formation (22, 23).

#### Vital NETosis

However, Clark et al. showed that NET formation does not always coincide with cell death (24), representing a distinct form known as vital NETosis. Upon neutrophil stimulation with *Staphylococcus aureus* and lipopolysaccharide-activated platelets, NET formation primarily occurred within 30 minutes, without accompanying neutrophil lysis (19). Platelets utilize Toll-like receptor 4 as a sensor for lipopolysaccharide (LPS) levels, initiating the formation of NETs once the infection surpasses a critical threshold (5). It provides an alternative pathway for NET production, characterized by its independence from the NOX complex. Consistent with the NO-independent NETosis pathway, activation of surface receptors opens SK3 channels, facilitating calcium influx, which in turn activates PAD4 (23). Subsequently, PAD4 collaborates with MPO and NE to induce chromatin decondensation. These chromatins aggregate with histones and other granular proteins, which are transported extracellularly by vesicles. Neutrophils can continue to perform chemotaxis, phagocytosis, and pathogen clearance after NET release (5).

In addition to the nuclear DNA contained in NETs, there may also be mitochondrial DNAs, produced by viable neutrophils. Mitochondrial NETosis was first described by Yousefi et al. in 2009, following granulocyte-macrophage colony-stimulating factor (GM-CSF) activation and subsequent stimulation with LPS or C5a (25). Using pharmacological and genetic methods to block ROS production showed that the formation of mitochondrial NETs is similar to suicidal NETs, as both depend on ROS (26). However, it is noteworthy that the release of mitochondrial NETs by neutrophils does not limit their lifespan and does not lead to cell death. So far, research on mitochondrial NETosis remains limited, highlighting the need for further exploration of its underlying mechanisms.

## ACTIONS OPERATED BY NETS DURING INFECTION

When considering the mechanisms by which the immune system isolates bacteria, NETs undoubtedly emerge as a compelling candidate. Once pathogens bind to the DNA networks, their sticky nature and immobilizing properties allow the entrapment of pathogens within a high-concentration anti-microbial protein environment, thereby facilitating extracellular bacterial eradication and inhibiting their colonization of new hosts (27). McDonald et al. showed that during endotoxemia and sepsis, neutrophils migrate to liver sinusoids and function by releasing NETs. Once NET formation is blocked, it impairs bacterial sequestration and enhances their dissemination to distant organs (28). So far, NETs have shown efficacy against a wide range of pathogens including bacteria, fungi, parasites, and even viruses.

One of the NET’s direct anti-microbial properties is due to DNA that sequesters surface-bound cations, disrupts membrane integrity, and lyses microbial cells. Treatment with excess cations or phosphatase enzymes neutralizes their anti-microbial activity, while the structure of the NETs, including the localization and function of the bound proteins, remains intact (29). Moreover, histones, as abundant components of NETs, possess distinct anti-microbial properties inherent to their individual subunits. A recent study shows that treatment of influenza A viruses with H4 results in decreased viral uptake and replication in epithelial cells (30). Virulence factors of *Salmonella* and *Yersinia*, especially *Shigella*, can be cleaved by NE when trapped in NETs. Notably, NE degrades *Shigella* virulence factors at concentrations 1,000 times lower than those needed to degrade other microbial proteins (31). Calprotectin (S100A8/A9) exerts broad-spectrum microbiostatic activity by strategically depriving essential Zn²^+^ and Mg²^+^ ions, effectively suppressing a range of fungal pathogens, including *C. albicans*, *Cryptococcus neoformans*, and *Aspergillus fumigatus* (32–34). Of course, NETs can restrict the transmission and infectivity of various parasites *in vitro*, such as *Entamoeba histolytica*, *Plasmodium falciparum*, and T*oxoplasma gondii*. However, in some cases, NETs only trap parasites or inhibit their growth without directly killing them (35). For example, *Schistosoma japonicum* can counteract the microbicidal effects through its soluble egg and larval antigens (36, 37).

The efficiency of NET components influences their effectiveness. Mice that are exposed to low levels of cathepsin G exhibit an increased susceptibility to infections from gram-positive bacteria like *S. aureus* (38). In contrast, those exposed to lower concentrations of elastase tend to be more vulnerable to infections caused by gram-negative bacteria, including *Escherichia coli* and *Klebsiella pneumoniae* (31). In spite of this, NETs have limited bactericidal properties. Azzouz et al. proved that NETs capture *P. aeruginosa* and *S. aureus* without directly eliminating them while promoting complement-mediated killing (39). In this study, it was also observed that under static conditions, NETs have limited bactericidal activity, whereas under dynamic conditions, their bacterial capture is enhanced, though bactericidal efficacy is reduced.

Mice deficient in NET production, especially those with gene deletions for key NET-associated proteins, are more susceptible to infections, highlighting the critical role of NETs in infectious diseases (40). Most studies on NET formation and their role in bacterial infections have been performed *in vitro*. In contrast, research on NET responses to bacterial infections *in vivo* remains limited. This gap is largely due to the technical challenges of validating NET function within living organisms, as well as difficulties in accurately quantifying variables such as stimulus type, dosage, and exposure duration (41).

## MODULATION OF NET RELEASE

The release of NETs is essential for the defense against pathogens. However, the action of NETs on bacteria is not sufficient for their death, as bacteria employ several mechanisms to minimize the anti-microbial NET effect and immunopathology. NET evasion appears to be a widespread survival strategy that allows pathogens to proliferate and spread (10), with multiple pathogens sharing common characteristics in how they modulate both the release and activity of NETs (36). Evasion strategies can be broadly categorized into three types: inhibition, resistance, and degradation, all of which underscore the remarkable resilience of pathogens in the presence of NETs.

### NET formation inhibition

The production of NETs is heavily reliant on the burst of ROS originating from both the cytosol and mitochondria. Consequently, numerous bacteria have evolved mechanisms to inhibit ROS-mediated NET formation. This suppression of ROS may be achieved through the disruption of ERK phosphorylation upstream of NADPH oxidase (42). For instance, the adenylate cyclase toxin produced by *Bordetella pertussis* generates supraphysiological levels of cyclic adenosine monophosphate, thereby impeding oxidative burst (42). The LPS component in the prevalent clone of *K. pneumoniae*, sequence type 258, has been shown to suppress ROS production, correlating with an increased survival rate (43).

In the host immune response, interleukin (IL)-8 functions as a crucial chemokine and activator for neutrophils, facilitating the formation of NETs. However, certain bacteria participate in the infection pathogenesis by inactivating the multifunctional host defense peptide, IL-8. For instance, the cysteine protease SpyCEP from group A *Streptococcus* (GAS) can cleave IL-8, while its functional homolog, CepI, found in the zoonotic pathogen *Streptococcus suis*, also targets IL-8, enhancing bacterial virulence (44). Additionally, GAS-secreted streptolysin O not only obstructs neutrophil degranulation and the secretion of IL-8 but is also both necessary and sufficient to block oxidative bursts, ultimately hindering NET formation (45). Alternatively, some pathogens induce the NET-suppressive cytokine IL-10 to block Toll-like receptor (TLR)-mediated response. Research has demonstrated that extracellular vesicles released by *Schistosoma* contain highly expressed Sja-miR-71a, which targets Sema4D, leading to the upregulation of IL-10 and subsequently inhibiting NET formation (46). Similar mechanisms have been noted in other pathogens like group B *Streptococcus* (GBS) (47) and HIV (48), which may enhance infection by dampening the immune response in specific circumstances. This implies that immunosuppressive cytokines may confer multiple advantages to the infecting pathogens.

Neutrophil surface receptors have been linked to NET formation. Human neutrophil Siglec-9 (hSiglec-9), a lectin, recognizes sialic acids (Sias) through its V-set immunoglobulin-like domain at the amino terminus and possesses a tyrosine-based inhibitory motif in its cytoplasmic tail (47). Pathogens expressing Sia, such as GBS, engage the inhibitory hSiglec-9, effectively dampening neutrophil activation and facilitating bacterial survival (49). Similarly, it has been observed that GAS expresses high-molecular-weight hyaluronic acid, which can also be specifically recognized by hSiglec-9, thereby inhibiting NET formation (10). Additionally, *Acinetobacter baumannii* enhances its infection process by suppressing the surface expression of CD11a on neutrophils, thereby impairing their adhesive function (50).

### NET resistance mechanisms

Besides, bacteria bolster their resistance by forming polysaccharide capsules or altering the electrochemical modifications of their surfaces. Capsules serve as virulence factors in certain microorganisms, enabling them to avoid being swallowed by immune cells, thereby enhancing bacterial pathogenicity (13). The composition, thickness, and physical properties of the capsule are involved in bacterial escape following NET capture (13). Notably, the expression of *Streptococcus pneumoniae* capsules (serotypes 1, 2, 4, and 9V) significantly reduces NET trapping without necessitating diminished susceptibility to NET-mediated killing (51). Research indicates that in primary pneumonia induced by *S. pneumoniae*, the production of NETs is closely correlated with the thickness of the bacterial capsule and proportional to the severity of the disease. Serotypes with thicker capsules exhibit greater resistance to NET capture and elimination (52).

Net-releasing proteins adhere to negatively charged phospholipids on the pathogen membrane through electrostatic affinity, thereby promoting the death of pathogens (53). Nevertheless, certain bacteria can modify their cell surface to diminish the binding affinity for NET-released anti-microbial proteins. This strategy is commonly observed in several gram-positive bacteria, such as *S. aureus* (54), *S. pneumoniae* (52), GAS (55), and *Listeria monocytogenes* (10). These bacteria activate specific operons that incorporate D-alanine into their peptidoglycan, thereby decreasing the negative charge of the cell wall. This adaptation enhances the bacterial ability to withstand the host’s immune responses and confers greater resilience against bile acids and antibiotics (13). In addition, *Neisseria meningitidis* spontaneously generates outer membrane vesicles that bind to NETs, saving *N. meningitidis* from NETs (53).

### NET component degradation

Exogenous and microbe-derived deoxyribonucleases (DNases) are the most potent inhibitors of NET function. Extracellular nucleases from certain bacterial species degrade the NET DNA backbone, compromising structural stability and facilitating immune evasion by eliminating NETs (10). For example, nucleases (Nuc) and extracellular adherence proteins produced by *S. aureus* interfere with the anti-microbial activity of NETs. Studies have shown that the production of *S. aureus* nuclease is associated with delayed bacterial clearance in the lungs and increased mortality following nasal infections (56). Moreover, *S. aureus* converts NET-derived nucleotides into deoxyadenosine via the action of adenosine synthase A, which triggers the caspase-3-mediated death of macrophages (57). The extracellular nuclease EndA secreted by *S. pneumoniae* counteracts NET capture mediated by the host, promoting bacterial spread from local sites to the lungs and eventually into the bloodstream, leading to increased bacterial dissemination and pathogenicity (58). It also secretes the nucleotide sequence-independent deoxyribonuclease TatD, which represents a novel potential extracellular nuclease that plays a crucial role in evading NET-mediated bactericidal activity (59). Similarly, *Mycoplasma pneumoniae* utilizes the nuclease Mpn491, which requires Mg²^+^ as a cofactor for its hydrolytic activity, to effectively degrade NETs (60). Once the DNA skeleton is broken down, the trapped bacteria are released. If not neutralized, opsonized, or cleared, they may persist in their ability to infect additional cells.

Bacteria can significantly compromise innate host defenses by orchestrating the degradation of various anti-microbial proteins within NETs. A prime example is the cysteine protease ApdS, derived from *S. suis*, which effectively cleaves LL-37. This enzymatic action results in a marked reduction in neutrophil chemotaxis while simultaneously inhibiting NET formation and ROS generation (61). Additionally, the fibronectin-binding protein B secreted by *S. aureus* functions as a predominant histone receptor, demonstrating a dual mechanism of immune evasion. It sequesters histones, obstructing their interaction with the bacterial membrane while also engaging plasminogen to facilitate its conversion to plasmin, ultimately disrupting the bound histone (62). Upon infection or stimulation, PAD4 is essential for the capture and elimination of bacteria via NETs. *Porphyromonas gingivalis* has been shown to exploit the properties of PAD4 to evade the host immune response. The bacterium produces *Porphyromonas* peptide arginine deiminase, which facilitates the escape from the lethal effects of NETs by inducing citrullination of histone H3 (63).

Interestingly, in cases where NETs fail to eliminate bacteria, their ability to capture certain microorganisms may paradoxically benefit pathogens. Some pathogens actively promote NET formation, subsequently employing nucleases to degrade the DNA scaffolds and utilizing the released phosphate groups as a nutrient source. It has been pointed out that the nuclease EddB secreted by *P. aeruginosa* degrades extracellular DNA, thereby serving as a nutritional resource for bacterial growth (64). Similar nucleases include those secreted by *S. aureus* (Nuc) (57) and *Vibrio cholerae* (Dns and Xds) (65), which promote the release of phosphate residues from DNA structures into the medium, thus facilitating nutrient acquisition and bacterial proliferation (13). While the mechanism by which pathogens utilize NETs as a nutrient source may exist in certain contexts, current evidence is insufficient to establish the universality of this mechanism across other types of pathogens. In summary, microorganisms modulate NETosis through diverse mechanisms, depending on their size and the expression of virulence factors.

## CROSSTALK BETWEEN NETS AND BOTH INNATE AND ADAPTIVE IMMUNITIES

**Fig 2:**
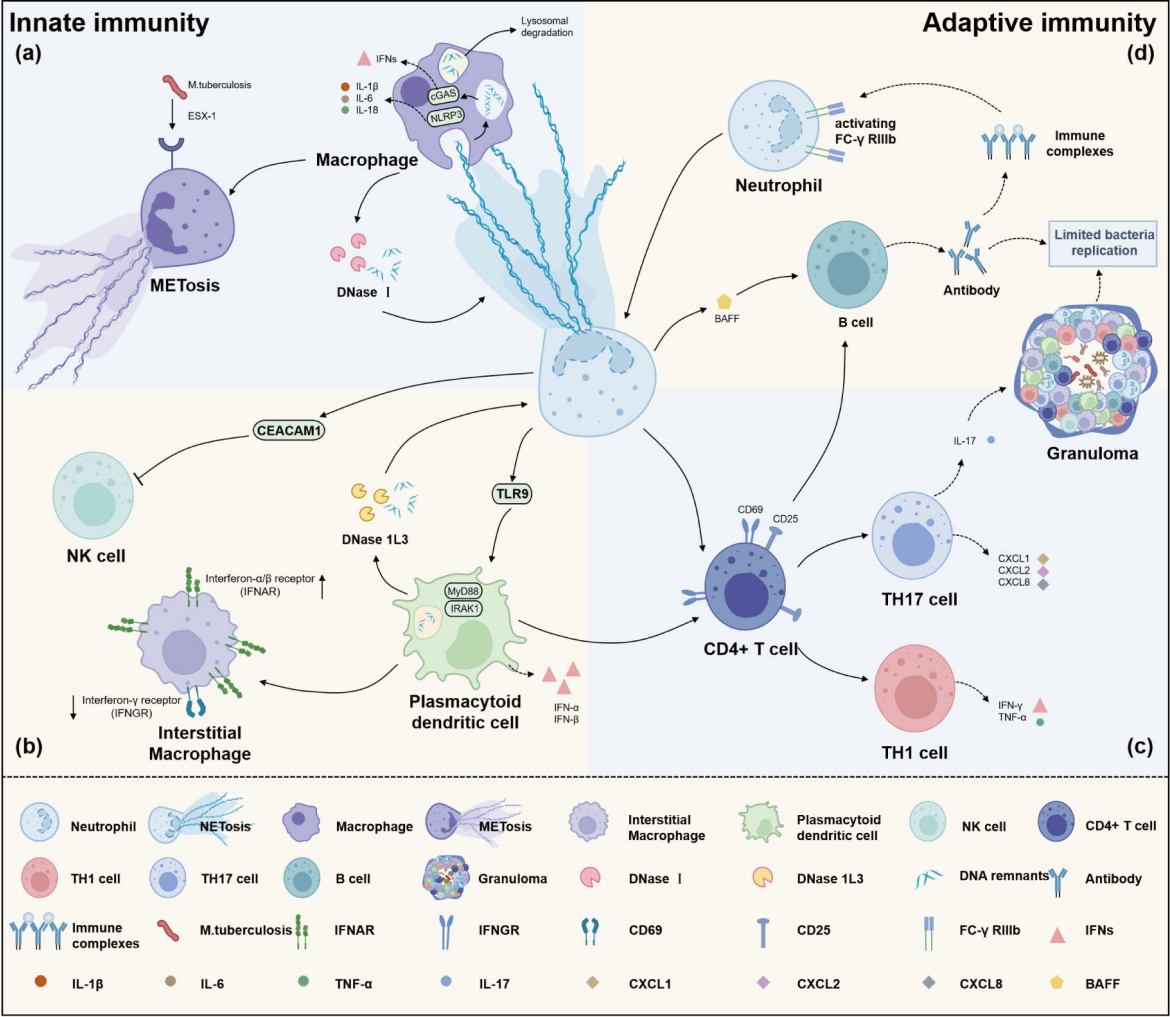
NETs regulate the immune microenvironment. NETs interact with innate and adaptive immunity during infection. (a) NET-derived substances activate cyclic guanosine monophosphate–adenosine monophosphate synthase (cGAS) and nucleotide-binding oligomerization domain-like receptor protein 3 (NLRP3) inflammasome in the cytosol of macrophages, triggering a series of proinflammatory cytokine releases. Meanwhile, the crosstalk between NETs and macrophages achieves optimal bactericidal activity through the internalization of NETs. Additionally, macrophages exposed to bacterial environments for extended periods can also form METosis structures. (b) NETs impair natural killer cell function via carcinoembryonic antigen-related cell adhesion molecule 1 (CEACAM1). Plasmacytoid dendritic cells are activated by DNA complexes in NETs from infected neutrophils, triggering type I interferon (IFN) (α and β) production while acting as a conduit between innate and adaptive immunity to activate T lymphocytes. (c) NETs can directly lower the activation threshold of T-cell responses and initiate the response of protective Th1 and T-helper 17 (Th17) cells. (d) NETs support B-cell activation via the B-cell activating factor (BAFF), and B-cell-derived immune complexes further activate neutrophils by binding to FC-γ receptor IIIb (FC-γ RIIIb), thereby inducing additional NET formation.

NETs extend beyond their direct anti-microbial function, as they contain substances like high-mobility group box 1 (HMGB1), the peptide LL-37, α-defensins, and DNA, all of which possess potent biological activity and function as classic danger signals. These components form DNA–protein complexes that serve as key indicators of cellular distress, triggering ligand–receptor interactions on innate immune cells and stimulating adaptive immunity (66). Such signals are recognized by neighboring or distant immune cells, including macrophages, natural killer (NK) cells, dendritic cells (DCs), and T and B lymphocytes, which possess endosomal and cytosolic receptors capable of initiating immune responses (67). NETs are not only integral to the anti-microbial response initiated by neutrophils but also significant in driving inflammation and orchestrating adaptive immune responses (Fig. 2).

### Interactions between NETs and macrophages

Neutrophils and macrophages are crucial to innate immunity, yet the mechanisms underlying their cooperative response to extracellular pathogens remain incompletely understood. Current research indicates that NET-forming neutrophils can activate macrophages through signaling pathways during the early stages of multicellular inflammation and granuloma formation (68). Macrophage-mediated inflammatory immune responses are involved in defense against bacteria (4). Upon translocation of NET-derived DNA into the cytosol of macrophages, cyclic guanosine monophosphate–adenosine monophosphate synthase is activated, leading to the production of type I interferons (IFN-Is) (69). This response also involves the activation of the nucleotide-binding oligomerization domain-like receptor protein (NLRP3) inflammasome, resulting in the release of proinflammatory cytokines, including IL-1β, IL-6, IL-8, IL-18, and tumor necrosis factor alpha (TNF-α) (70, 71), which activate T-helper 17 (TH17) cells and amplify immune cell recruitment, especially in atherosclerotic plaques (72). However, prolonged exposure to NETs or their components apparently triggers macrophage cell death via caspases and apoptosis-inducing factor, likely due to a decrease in mitochondrial membrane potential (73).

Indeed, NETs serve as a powerful and effective weapon against bacterial infections by functioning as pseudo-opsonins that enhance macrophage phagocytic activity (71). Monteith and colleagues demonstrated that in the context of bacterial infections, NETosis does not directly augment the bactericidal activity of neutrophils. Instead, it improves overall anti-microbial defense by facilitating macrophage phagocytosis and the transfer of specific anti-microbial peptides (AMPs) from neutrophils to macrophages (7). Within macrophages, these AMPs regain their full anti-microbial efficacy upon engulfment into the phagosome (71). This process has been corroborated across various bacterial pathogens, including *S. aureus*, *S. pneumoniae*, and *P. aeruginosa*, suggesting its widespread role in boosting anti-bacterial immunity. In a similar manner, apoptotic neutrophils can also be a source of AMPs for macrophages, influencing cytokine profiles and enhancing the ability to effectively eliminate intracellular bacteria (74).

Beyond their roles in antigen presentation and phagocytosis, macrophages fine-tune the formation and removal of NETs. Under normal physiological conditions, NETs are promptly cleared, along with pathogens, preventing sustained inflammation (71). Haider et al. examined the ability of various macrophage subsets to degrade NETs both *in vitro* and *in vivo*, using a murine thrombosis model. Macrophages cultured on blood clots or isolated NETs and subsequently polarized were able to degrade NETs through DNase I digestion and opsonization by C1q (75). When the physiological concentration of extracellular DNase I is insufficient to completely clear the NETs, macrophages engulf the remnants, which are internalized into vesicles that fuse with lysosomes for degradation of NET components and encapsulated pathogens (76). Furthermore, the cytoplasmic exonuclease TREX1 (DNase III), rather than DNase II, is involved in NET degradation. It is noteworthy that the macrophage-mediated clearance of NETs occurs without eliciting an inflammatory response, thereby acting as an immune-silencing mechanism (77).

A subsequent study revealed that exposure to NETs induced a proinflammatory response in M2 macrophages, whereas M1 macrophages underwent cell death accompanied by nuclear decondensation, leading to an increase in extracellular DNA, likely representing METs (78). Wong and Jacobs were the first to report that macrophages are involved in *Mycobacterium tuberculosis*-induced extracellular trap release, a process that occurs in an esx-1-dependent manner (79). This phenomenon has since been confirmed in primary macrophages as well (80). However, METs may also provide a niche for bacteria to survive and aggregate under certain conditions. Je et al. discovered that the released METs have no bactericidal activity against the *Mycobacterium* mass they entrapped (81). While NETs and METs serve to immobilize pathogens, their microbicidal effects are context dependent.

### NETs impair NK cell function

NK cells are innate cytotoxic lymphocytes that target tumor cells and pathogens. Upon activation, they release perforin and granzymes to execute their cytotoxic functions (76). However, the function of NK cells is known to be primarily inhibited by NETs. Studies have shown that the infusion of NET-degrading enzymes (DNase I) combined with NK cells into postoperative hepatocellular carcinoma (HCC) patients significantly reduces the risk of recurrence after HCC resection, with no notable systemic toxicity observed (82). These findings suggest that NETs may suppress NK cell activity through specific mechanisms and that the application of DNase I effectively mitigates this suppression, thereby enhancing the anti-tumor efficacy of NK cells. Analysis of NET-related genes from RNA-Seq data of coronavirus disease 2019 (COVID-19) patients revealed that NETs may inhibit NK cell function via carcinoembryonic antigen-related cell adhesion molecule 1, a known negative regulator of NK cells (83). This suppression could weaken the host’s anti-microbial immune response, leading to an imbalance in immune defense. Moreover, similar to other immune models, the disruption of NET formation in this context might significantly alter NK cell activity (84), suggesting a potential therapeutic target for modulating immune responses in viral infections like COVID-19.

### NETs mediate modulation of dendritic cells

IL-4 and GM-CSF are well-established inducers of human monocyte differentiation into DCs. Guimarães-Costa et al. described that NETs interfere with the differentiation process driven by IL-4 and GM-CSF, reprogramming the development of monocyte-derived dendritic cells into an anti-inflammatory macrophage phenotype, thereby impairing their ability to effectively combat pathogens (85). However, co-culturing LPS-stimulated neutrophils (LPS-polymorphonuclear neutrophils [PMNs]) with immature dendritic cells (iDCs) drives DC maturation, as evidenced by the upregulation of CD40 and CD86, along with the secretion of IL-12 and TNF-α (86). In conclusion, this implies that NETs can modulate DC function.

iDCs recognize microorganisms or danger signals in their microenvironment mediated by high levels of innate receptors, such as TLRs, C-type lectins, complement receptors, Fc receptors, heat shock proteins, Nod-like receptors, and scavenger receptors. Upon activation, DCs enhance the expression of MHC class II molecules and co-stimulatory molecules (e.g., CD80, CD86, and CD40) while also altering their cytokine secretion profiles (86). Notably, LL37 and HMGB1 present in NETs promote the uptake and recognition of self-DNA by plasmacytoid dendritic cells (pDCs) (87). Specifically, the complex of LL37 and HMGB1 with self-DNA binds to TLR9 on the surface of pDCs and activates the transcription factor IRF7 via the MyD88-IRAK-1 pathway, resulting in significant IFN-I release (88). In patients with systemic lupus erythematosus (SLE), NETs have been shown to be the dominant factor driving IFN-I production by pDCs (87).

Research has shown that bacteria do not directly infect pDCs or induce IFN-I secretion. Instead, pDCs are activated by DNA complexes in NETs from infected neutrophils, triggering IFN-I (α and β) production (89). While bacterial infection stimulates NET formation, this alone is insufficient to induce IFN-I production in pDCs, suggesting the involvement of additional factors. During *M. tuberculosis* infection, IFN-I produced by stromal macrophages and pDCs interferes with the IFN-γ response, impairing bacterial control. This creates a positive feedback loop of extensive NET formation and IFN-I production, further amplifying neutrophil recruitment and advancing active pulmonary tuberculosis (90). Meanwhile, DCs, as key antigen-presenting cells like macrophages, can degrade NETs via DNase1L3 (77). Removal of pathogens also helps maintain NET homeostasis, thereby preventing excessive tissue and organ damage. Unexpectedly, prolonged exposure to NETs resulted in DC death, with ultrastructural analysis revealing mitochondrial changes, as did macrophages (73).

Net-activated DCs serve as a key bridge between innate and adaptive immunity. Sangaletti and colleagues reported that upon co-culturing myeloid dendritic cells (mDCs) with PMNs, mDCs predominantly interact with NETs, with minimal engagement observed with necrotic neutrophils. Confocal immunofluorescence analysis further corroborated that mDCs can load with NET components, including extracellular proteins and DNA (91). It can be proven that DCs are capable of processing NET-derived antigens for subsequent antigen presentation. The presented antigens are recognized by T lymphocytes, eliciting a robust protective Th1 response and orchestrating the secretion of IFN-γ and IL-17 (4, 92). Remarkably, exposure of myeloid DCs to NET components induces anti-neutrophil cytoplasmic antibodies and autoimmunity when injected into naïve mice, underscoring the strong immunogenicity of NET structures and their potential to trigger adaptive immune responses (91).

### NETs regulate T-cell responses

Dendritic cells present exogenous antigens to T lymphocytes, initiating T cell-mediated immune responses, which subsequently promote the formation and maturation of granulomas. In the initial stages of granuloma formation, it promotes the accumulation of immune cells at the site of infection, thereby fostering localized immune responses and the containment of pathogens (93). For example, a genetically engineered zebrafish model showed that neutrophils are recruited to nascent granulomas via signals from dying infected macrophages, where they phagocytose the bacteria (94). In the absence of CD4 Th1 cells, *M. tuberculosis* can disseminate despite intact granuloma structure (93), meaning that effective control of bacterial infection necessitates the involvement of antigen-specific CD4+ T cells. Extracellular histones on NETs have been shown to trigger an upregulation of IL-17 and Th17 responses (95), especially in periodontal pathogen-mediated NETs like *P. gingivalis* (96) and *Actinobacillus actinomycetemcomitans* (97). Th17 cells, another key effector CD4+ T-cell subset, synthesize cytokine IL-17, which enhances the activity of bactericidal peptides, including LL-37 and β-defensins. They also synthesize chemokines CXCL1, CXCL2, and CXCL8, inducing the influx of immunocompetent cells into inflammatory foci (66). In mice with IL-17A gene knockout, they fail to form mature granulomas and impair the protective response in the lungs infected with *M. bovis* bacille Calmette–Guérin (98). Thus, the activation of Th17 cells is imperative for bolstering immunity against pathogen infection.

Besides, NETs can directly lower the activation threshold of T-cell responses to specific antigens and even to suboptimal stimuli through upregulating CD69 and CD25 on them (99). This certainly demonstrates a new link between innate and adaptive immune responses. However, PD-L1 carried by neutrophils and NETs suppresses T-cell function *in vitro* and *in vivo*. A study shows that the blockade of PD-L1 with anti-PD-L1 antibodies alleviates lymphocyte depletion induced by sepsis, significantly improving survival rates in murine models (100). Surprisingly, NETs capture not only pathogens but also CD4+ T cells, CD8+ T cells, B cells, and monocytes under certain circumstances, potentially resulting in indiscriminate loss of systemic immune cells. For instance, in non-human primates infected with SIV/HIV, CD4+ T-cell depletion occurs due to the entrapment of immune cells by NETs, which inflict lethal damage characterized by membrane rupture and nuclear disintegration, regardless of the presence of infection (101).

### NETs in B cell-mediated antibody production

Neutrophils collaborate with B cells to form an innate anti-microbial immunoglobulin defense, providing a rapid and non-specific anti-bacterial mechanism. Puga et al. showed that neutrophils in the spleen’s marginal zone (distinct from circulating neutrophils) secrete B-cell activating factor (BAFF), APRIL, and IL-21, which activate MZ B cells to induce class switching, somatic hypermutation, and antibody production (102). Transcriptomic analysis of NETs produced by neutrophils in COVID-19 patients further confirmed that these structures also activate B cells through BAFF (83). Moreover, immune complexes from B cells can activate neutrophils by binding to the FC-γ receptor IIIb, triggering additional NET formation (92). This intricate immune network not only defends against pathogens but also involves multiple feedback loops that contribute to the onset of autoimmune responses and disease progression.

## ADVERSE EFFECTS OF NETS

As for all effective immune responses against pathogens, recent *in vitro* and *in vivo* experiments have demonstrated that the molecular cascades involved in NET formation confirm their accompanying deleterious effects. In severe infectious diseases, the “dysregulation” of neutrophils can induce their migration to the locus of infection, which may lead to the elimination of bacteria while causing other disasters (103). In particular, when host mechanisms for clearing and degrading NETs are overwhelmed, these structures begin to accumulate in the bloodstream, becoming detectable in serum. This review primarily focuses on the harmful effects of pathogen-induced NETosis in infectious diseases (e.g., sepsis and COVID-19) and the various systemic pathologies that may arise (e.g., autoimmune diseases and thrombotic disorders). Below, we will adopt infectious diseases as the central paradigm while contextualizing parallels in non-infectious pathologies to dissect the harmful effects of NETs.

### NETs exaggerate inflammation

NET-derived products (e.g., LL37, HMGB1, histones, and DNA) could serve as DAMPs activating caspase-1 initiating NLRP3 inflammasome activation in macrophages, resulting in the release of IL-1β and IL-18. In turn, these cytokines also stimulate further NET formation, creating a feed-forward inflammatory loop (104). In the early phases of sepsis, neutrophils are mobilized from the bloodstream to the site of infection, where they release NETs (105). Research has demonstrated that impaired NET function during this initial infection phase plays a crucial role in sustaining systemic inflammation that initiates the onset of sepsis. Importantly, NET levels in infantile sepsis patients are higher than those in adults, and the severity of sepsis correlates positively with NET levels. It is likely due to the elevated production of inflammatory cytokines that promotes neutrophils’ accumulation (103). While this feed-forward loop similarly occurs in viral pathologies, for example, severe acute respiratory syndrome coronavirus 2-induced NETs exacerbate alveolar inflammation in COVID-19 (106), underscoring the universality of NET-driven immunopathology across pathogens.

### NETs cause organ damage

Sepsis represents a condition characterized by lethal dysfunction of multiple organs resulting from a dysregulated immune response to infection and is associated with high morbidity and mortality rates (107). In sepsis and acute injury, free-circulating proteases and granular proteins cause cytotoxicity to endothelial cells due to their ability to compromise cell membrane integrity (108, 109). The direct destructive effect of NET components, in conjunction with the inflammatory environment and oxidative stress, degrades the glycocalyx on the surface of endothelial cells and increases permeability. The collapse of the endothelial barrier promotes microvascular leakage, leading to vascular hypotension, tissue edema, and fatal organ failure (107). A prime example, NETs are linked to hepatic damage during sepsis caused by methicillin-resistant *S. aureus*, and this damage can be averted by deficiency in NE or PAD4 (41). The COVID-19 pandemic has highlighted the generation of NETs correlated with exaggerated inflammation and damage to the alveolar capillary barrier (110). Markers of NET formation were elevated in the sera of COVID-19 patients compared to healthy donors (107, 111). The aggregation of DNA strands into large complexes can also lead to local obstruction of the small bronchus (112). Notably, the levels of these markers significantly declined during the recovery phase of the disease. Thus, NETs are regarded as pathological because they accumulated in areas of vital organs, such as the lung, kidney, and liver (103). However, the mechanism by which the infiltrating NETs damage these vital organs is obscure.

### NETs trigger thrombosis formation

Thrombosis formation occurs due to the obstruction of normal blood flow by blood clots in the arteries or veins. This dynamic pathological process begins with a synergistic interaction between activated neutrophils, platelets, and coagulation factors (113). Activated platelets can recruit neutrophils to the site of infection and release NETs when sepsis develops (113). NETs further promote platelet adhesion, activation, and aggregation and exert procoagulant activity, thereby providing a scaffold for thrombosis (105). In mouse models of sepsis infection, histone release in NETs is thought to be a key factor leading to intravascular thrombosis (108). McDonald et al. demonstrated in an *in vivo* infection model that the dynamic NET–platelet–thrombin axis promotes intravascular coagulation and microvascularization in sepsis (114). Accumulated NETs are conducive to sustaining excessive immune-driven thrombosis during sepsis, ultimately resulting in fatal disseminated intravascular coagulation complications in affected patients (113). In addition, the use of intercellular adhesion molecule 1 inhibitors to inhibit neutrophil homing to endothelial cells significantly reduced thrombus formation in mice (115). Platelet factor 4 binds to NETs to resist DNase, thereby promoting microvascularization, a process thought to be a key factor in the development of acute respiratory distress syndrome in patients with COVID-19 (116). Therefore, the relationship between NETosis and thrombosis is likely bidirectional. This interconnected cascade may be crucial in conditions like thrombophilia, particularly in patients with thrombosis or those at heightened risk of clot formation following an infection.

### NETs drive autoimmune reaction

The hallmark of autoimmune diseases is the loss of immune tolerance, which results in an aberrant enhancement of the immune system’s ability to recognize and attack self-antigens, thereby initiating self-directed immune responses (117). NET overflow drives an autodestructive process as many substances associated with NETs have been identified as neoself-antigens (22). Accordingly, NETs have been implicated in systemic pathology associated with disease entities including but not limited to rheumatoid arthritis, SLE, and anti-neutrophil cytoplasmic antibody-associated vasculitis (113). For example, SLE patients show decreased DNase activity and NET degradation, which may be alleviated by DNase I therapy (92). In individuals with deficiencies in enzymes responsible for degrading NETs, even pathogens that typically have a minimal effect on NET formation can contribute to systemic pathology linked to NETs. It represents a potential mechanistic connection between pathogen infection and the development of systemic autoimmune diseases. This hypothesis is further supported by the observation that viral infections frequently lead to the production of autoantibodies, which are known to not only mimic the pathophysiology of SLE but also initiate its onset or exacerbate disease flares (118).

These pathological effects well explain why NETs have a double-edged sword effect when it comes to resisting pathogen invasion. NETs deposited in infected areas can effectively act as disease-resistant agents and protect the host from damage. However, when NETs are deposited in large amounts in healthy tissues, they may have serious consequences for the host, triggering systemic pathological reactions (22). Consequently, targeted strategies to regulate NET formation at pertinent disease stages could offer promising therapeutic avenues for managing infections more effectively.

## CONCLUSION

Neutrophils as central cells of the innate immune system establish a robust immune defense by phagocytosing pathogens and secreting anti-microbial factors. The discovery of NETosis unveiled a novel and distinctive immune defense mechanism in which neutrophils release a web-like structure to capture and neutralize pathogens (18). Upon infection, neutrophils release a cascade of inflammatory mediators along with NETs, further recruiting additional immune cells to the site of infection and establishing a focal point for the immune response. The role of NETs extends beyond the innate immune system, influencing the initiation and regulation of adaptive immunity through interactions with T and B lymphocytes. By exposing immune-related molecules and cytokines, NETs can enhance immune responses or, in certain contexts, suppress them. For instance, NETs may modulate antigen presentation by dendritic cells, thereby impacting T-cell activation and immune tolerance (99). The immunomodulatory properties of NETs are essential for orchestrating an appropriate inflammatory response, as well as limiting excessive inflammation to maintain homeostasis. However, NETs function as a double-edged sword, offering fundamental anti-microbial defense while also simultaneously promoting tissue damage and inflammation in various diseases (113). Excessive deposition of NETs near infected areas may exert cytotoxicity, causing considerable collateral tissue damage (22). Consequently, NETs represent more than a singular mechanism of pathogen clearance, and they play multifaceted roles in inflammation and immune homeostasis. The immune response governed by NETs may manifest additive, synergistic, or antagonistic interactions, depending on the pathological context, ultimately shaping both its intensity and direction. Comprehending the dynamic regulation of NET levels to sustain homeostasis will aid in the formulation of more holistic clinical treatment strategies and be pivotal for guiding future research.
